# Factors Governing the Cross-Species Virulence of Shiga Toxin-Producing *Escherichia coli*

**DOI:** 10.3390/pathogens15040353

**Published:** 2026-03-26

**Authors:** Paul Hart, Alexander Bowitch, Alexander Mellmann, Denise M. Ferkey, Gerald B. Koudelka

**Affiliations:** 1 Department of Biological Sciences, University at Buffalo, 109 Cooke Hall, Buffalo, NY 14260, USA; pbhart@buffalo.edu (P.H.); zander.bowitch@gmail.com (A.B.); dmferkey@buffalo.edu (D.M.F.); 2 National Consulting Laboratory for Hemolytic Uremic Syndrome (HUS), Institute of Hygiene, University Hospital Münster, 48149 Münster, Germany; alexander.mellmann@ukmuenster.de

**Keywords:** Shiga toxin, bacteriophage, STEC, predation, anti-predator defense, toxicity, *Acanthamoeba castellanii*, *Caenorhabditis elegans*

## Abstract

Phage-encoded Shiga toxin (Stx) released by Shiga toxin-producing *E. coli* (STEC) can kill multiple eukaryotic bacterial predators, including *Acanthamoeba castellanii*, *Tetrahymena thermophila* and *Caenorhabditis elegans*. However, the impact of Stx type, Stx amount, and the serogroup of the *E. coli* on the effectiveness of this exotoxin are poorly understood. These factors impact the severity of Stx-mediated disease in humans and therefore, by studying their role in modulating predator–prey interactions, we may gain insight into how these virulence factors evolved to contribute to human pathogenicity. Herein, we investigated the effects of these factors on predator killing by measuring the efficiency with which five different hemolytic uremic syndrome (HUS)-causing STEC strains consume and/or kill *A. castellanii* and *C. elegans*. These strains express various combinations of Stx types and amounts and O-antigens. We found that variations in Stx types and amounts significantly affect the ability of a given bacterial strain to kill predator *A. castellanii* and *C. elegans*, with higher Stx1 titers (HUSEC 31 vs. 19) and the presence of Stx2 alone (HUSEC 20) correlating with significantly greater predator killing. These attributes also affect STEC pathogenicity in humans, suggesting that ecological selective pressures for anti-predator defense inadvertently drive the evolution of strains with higher virulence potential in humans.

## 1. Introduction

Shiga toxin (Stx)-producing *Escherichia coli* (STEC) are foodborne pathogens of emerging public health importance. STEC are estimated to cause more than 265,000 illnesses each year in the United States alone [1]. STEC cause diarrhea, hemorrhagic colitis (bloody diarrhea), and the potentially fatal hemolytic uremic syndrome (HUS) [2].

STEC comprise a very large genetically and phylogenetically heterogeneous group of organisms [3]. They are found in water, soil, on food crops, in both raw and processed foods [4,5], and in the intestinal tracts of domesticated and wild animals, as well as in humans [6,7,8]. The most commonly identified disease-causing STEC strain is serotype O157:H7, which accounts for ~50% of all STEC infections world-wide [9]. However, several non-O157 STEC serogroups (including O26, O45, O103, O111, O121, and O145) also cause severe illness and death in humans [10,11]. One study isolated 71 different non-O157 STEC serogroups from humans, but not all were associated with human disease [12], suggesting that O-antigens may play a role in regulating disease and/or disease severity.

Phylogenomic analyses suggest that STEC O157:H7 strains found worldwide are highly related [13]. However, not all STEC O157:H7 strains are equally capable of causing severe disease in humans [14,15]. Likewise, there are within-serogroup differences in the severity of illness caused by non-O157 STEC serogroup strains, even among strains that are highly related to each other [16]. The reasons for the variation in the severity of STEC-mediated disease within and/or between STEC serogroups are unclear. However, it is known that host factors (e.g., demographics, immune status) play a role in modulating disease severity [17,18,19,20].

The essential virulence factor of STEC is Stx toxin. Stx is a ribosome-inactivating protein that kills eukaryotic cells by cleaving a specific adenine base in the of the sarcin–ricin loop of large ribosomal RNA (rRNA). This effectively halts protein synthesis, a process crucial for cell survival, leading to cell death and severe disease in humans [21,22,23,24]. STEC occurs through the infection of benign or mildly pathogenic *E. coli* strains with temperate bacteriophages that encode this toxin [13,25,26,27,28,29]. All STEC strains carry at least one Stx-encoding bacteriophage, which may encode either of two types of this toxin: Stx1 or Stx2. These types are distinguished by their biological activity and sequence [30,31]. A variety of subtypes of Stx1 (1a, 1c, 1d and 1e) and Stx2 (2a-o) have been identified [30,31]. These types and subtypes vary in their host distribution characteristics and biological activities, but all Stx subtypes have been found in STEC-infected humans [32]. While Stx1 and Stx2 types are found equally frequently in STEC-related human infections [33], specific Stx subtypes, including Stx1a, Stx2a, 2b, 2c, and 2d, are more commonly associated with human disease. In contrast, Stx subtypes 1c, 1d and 1e are rarely associated with severe human disease [30]. Similarly, Stx2e-o are infrequently associated with severe human disease, but more research on these subtypes is needed [3].

Sequence analysis indicates that genomic variations in a given STEC strain are primarily due to differences in Stx prophage carriage and Stx prophage gene content [34,35]. In addition, identical Stx-encoding prophages are found in both STEC O157:H7 and non-O157 STEC, including an Stx2-encoding phage that was associated with a high incidence of HUS [35]. These observations suggest that additional phage-encoded factors play a role in regulating STEC virulence.

The gene regulatory patterns and life cycles of Shiga toxin (Stx)-encoding phages are highly similar analogous to non-toxigenic lambda-like bacteriophages. Upon host infection, these lambdoid phages undergo a binary developmental choice: the lytic cycle, resulting in host cell death, or lysogeny, where the prophage integrates into the host genome and remains quiescent [36,37,38]. Critically, *stx* expression is functionally coupled to the lytic cycle; high-level toxin production and its subsequent extracellular release are dependent on phage-mediated host cell lysis. Consequently, the molecular “switch” governing the transition from lysogeny to lytic induction is the primary determinant of Shiga-toxigenic *Escherichia coli* (STEC) virulence.

There are an overwhelming amount of phage-encoded exotoxin genes in the environment [39]. The prevalence of these genes far exceeds the number of potential human targets. Moreover, phages that encode exotoxins are often found in environments where humans and other mammalian targets are not prevalent [40]. These observations suggest that exotoxins are not specifically targeted towards mammals [41]. Instead, they may have first evolved as part of an anti-predator defense strategy, with humans simply being innocent bystanders in the microbial war between protist predators and bacterial prey [42,43,44,45,46,47].

Consistent with this idea, most phage-encoded exotoxins kill eukaryotic cells by acting on pathways that are universally conserved across eukaryotes. As discussed above, Stx toxin kills eukaryotic cells by inactivating the ribosome via deglycosylation of the large subunit rRNA in the sarcin–ricin loop [48]. This region is universally conserved, present in large ribosomal subunit RNAs across the evolutionary spectrum, ranging from bacteria, single-celled eukaryotes and invertebrates to both simple and complex vertebrates, including mammals. Hence, the ribosomes of all these organisms are susceptible to being inactivated by Stx.

Further supporting this idea, bacteria that contain a phage known to produce exotoxin Stx2 have a survival advantage against the protist bacterivores *Tetrahymena thermophila* [49] and *Acanthamoeba castellanii* [50]. Similarly, STEC also impede the growth of the bacterivorous worm *C. elegans* [51,52,53].

Given these observations, we hypothesized that if Stx evolved as an anti-predator defense, then variations in Stx type, titer, and bacterial surface structures that affect predator interactions would correlate with differential killing of bacterivores. Here, we test this hypothesis by examining the effects of these factors on the survival of two evolutionarily distinct predators, *A. castellanii* and *C. elegans*. Because of their prevalence in HUS cases, we chose to examine the effect of Stx1 and Stx2 on predator survival. To test the applicability of our findings with single-celled protists to multicellular organisms, we also extended our studies to examine the effect of these STEC attributes on the killing of the bacterivore *C. elegans*.

Here we show that variations in Stx type and titer affect the ability of a given strain to kill its predators. Importantly, the effects of the variations in these attributes on the efficacy of bacterivore anti-predator defense correlate to differences in human pathogenicity.

## 2. Materials and Methods

### 2.1. Strains

*A. castellanii* was a gift from Wendy Trzyna, Marshall University (Huntington, WV, USA). The *C. elegans* laboratory wild-type strain N2 (Bristol, England) was obtained from the *Caenorhabditis* Genetics Center (CGC), which is funded in part by the National Institutes of Health—Office of Research Infrastructure Programs (P40 OD010440). Due to different predator growth requirements, different non-phage, non-Stx-expressing bacterial strains were used as negative controls for feeding experiments performed with *A. castellanii* and *C. elegans*. For *A. castellanii*, the *E. coli* K12 strain MG1655 [54] was obtained from the *E. coli* Genetic Resource Center (https://ecgrc.net/). For *C*. *elegans*, the uracil-requiring mutant strain OP50 of *E. coli*, obtained from the *Caenorhabditis* Genetics Center, was used.

Worms were maintained at 20 °C on NGM agar plates seeded with OP50 *E*. *coli* bacteria [55]. *A. castellanii* were maintained as described previously [56] (see also below). To discern the independent effects of Stx type, Stx amount, and O-antigen, we selected five strains from the HUSEC collection that allowed for the following key comparisons: (i) high vs. low Stx1 titer in an OR background (HUSEC 31 vs. 19); (ii) Stx2-only vs. Stx1 + Stx2 vs. Stx1-only in an O26 background (HUSEC 20 vs. 14 vs. 13); and (iii) effect of O-antigen in strains with similar Stx1 titers (HUSEC 31 vs. 13). HUSEC strains were isolated from fecal samples of epidemiologically unrelated patients with HUS (1 strain per patient) as described in [57], using procedures described therein. HUSEC serotypes were determined by using antisera against *E. coli* O-antigens 1–181 and H antigens 1–56. The type and amount of Stx produced by the individual HUSEC strains was determined as described in [57,58,59] (Table 1).

### 2.2. Cultivation and Harvesting of A. castellanii

*A. castellanii* were grown in 5 mL ATCC medium: 712 PYG w/additives at 30 °C in flat-bottom 25 mL tissue culture flasks. After 4 days of stationary incubation, growth media were decanted from flasks and 5 mL fresh medium was added to each flask. Flasks were placed on ice for 20 min to displace amoeba from the bottom of the flask and cells were then collected for passage or experimentation. Stocks of *A. castellanii* were made and stored at 4 °C as described [60]. *Acanthamoeba* were prepared for analysis by centrifugation at 300× *g* at room temperature, followed by three washes with PAS (Page’s Amoeba Saline Solution) [61,62] and then suspended in PAS. Amoebae cell counts were determined using a LUNA-FL™ Dual Fluorescence Cell Counter (Logos Biosystems, Annandale, VA, USA).

### 2.3. Amoeba Predation Assay

The predation assay was performed as described in [50]. Briefly, bacteria were grown to saturation in LB. Multiple proteose peptone glucose (PPG) agar plates were separately seeded with 100 μL of saturated cultures (~10^9^ cells/mL) of different bacterial strains. Initially, a portion of the bacteria was plated on LB agar plates to determine the numbers of cells present in the saturated cultures of each strain. This was found to be highly reproducible. Identical numbers of the different strains were plated on these separate plates. After incubation for 1 h at 37 °C, 10 μL of PAS containing 10^5^ *A. castellanii* trophozoites was spotted on each plate. Plates were sealed with parafilm and incubated at 28.5 °C. After 5 days, the surface area cleared by the amoeba on each plate was measured and recorded. The normalized plaque size is directly related to the amounts of bacteria consumed by the amoeba in the 5-day span, giving a measure of how successful each of the bacterial strains is in resisting amoeba predation. The data shown represent ≥3 independent biological replicates, with each biological replicate consisting of four technical measurements.

### 2.4. Amoeba Killing Assay

The amoeba killing assay was performed as described in [50,56,63]. Briefly, 400 µL of freshly harvested 4–6 days old amoebae was aliquoted into each well of a 24-well polystyrene tissue culture plate and incubated at 30 °C overnight. After removing the planktonic cells suspended in medium, the amoeba cells adhering to the bottom of each well were washed at room temperature three times with PAS. HUSEC bacteria were prepared by growing in LB overnight at 37 °C; 1.5 mL of bacteria was harvested and washed at room temperature three times at 3400× *g* with PAS. The bacteria were resuspended in 1 mL PAS. Repeated control experiments performed with all strains established the number of viable bacteria present after processing. This was done to ensure equal numbers of bacteria were subsequently incubated with amoeba.

Subsequently, 250 µL of a 1:10 dilution of the washed bacteria was added to each well of the 24-well plate. A portion of the washed bacteria was reserved and plated to confirm input cell counts. After bacterial infection and incubation, a mixture of acridine orange/Propidium Iodide stain was added to each well and the amoebae were released from the plate by incubating on ice. The total number of amoebae and the percent of live cells in each sample were measured by a LUNA-FL™ Dual Fluorescence Cell Counter (Logos Biosystems, Annandale, VA, USA) as described by the manufacturer. The results are expressed as the percent increase in dead amoeba cells in the presence of STEC bacteria relative to the number of such cells incubated in culture medium alone (i.e., (% dead in experimental − % dead in control)/% dead in control × 100). Three technical replicates were measured and averaged. The data shown represent ≥3 independent biological replicates with each biological replicate consisting of three technical measurements.

### 2.5. C. elegans Survival Assay

*C. elegans* survival/lifespan assays were performed essentially as previously described [64,65,66]. Briefly, NGM agar plates [55] were seeded with 50 µL of an overnight bacterial culture and left to incubate at 30 °C overnight. After plates had acclimated to room temperature (20–21 °C), L4-stage *C. elegans* were added to each plate in replicates of 15–20 worms and moved to a 20 °C incubator. Each plate was checked for dead worms every 12 h and during the first five days the surviving adult worms were also passaged to new plates every six hours to prevent offspring from confounding results. Dead worms were identified by verifying that pharyngeal pumping had ceased and further verified by touching the worms with a platinum wire pick to ensure that it did not elicit movement. Missing worms were subtracted from the starting count of worms. The survival time of the worms was quantified by measuring the restricted mean survival time (RMST) [67]. RMST quantifies the average lifespan of an organism and is independent of any assumption of proportional hazards. These values were calculated using OASIS (Online Application for Survival Analysis) [68]. Except where indicated, all the RMST value comparisons are significant at *p* ≤ 0.02.

## 3. Results

To begin to investigate the effects of Stx titer, Stx type, and serogroup on STEC strain virulence, we examined the ability of five STEC strains to act as an anti-predator defense against *A. castellanii* predation (Table 1). These strains are known to cause severe disease in humans; each of these strains was isolated from a patient with HUS and are part of the HUSEC Collection [57]. Thus, as a control we compared the effects of these strains on *Acanthamoeba* viability to that of MG1655, a laboratory strain of *E. coli* that encodes no known exotoxins.

**Figure 1:**
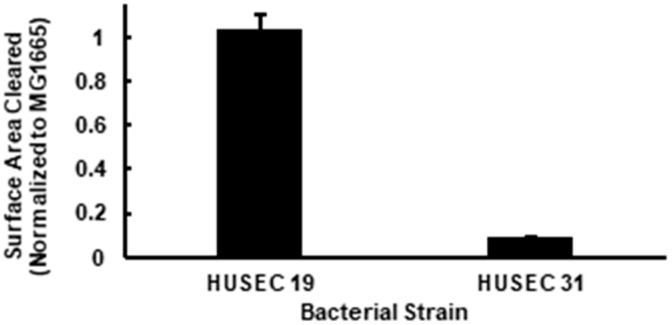
Normalized values for plaque surface area formed by *A. castellanii* on lawns of HUSEC 19 and HUSEC 31. PPG plates seeded with both strains were measured after five days of growth. Both HUSEC 19 and HUSEC 31 are OR and Stx1 strains with different induced Stx titers, as shown in Table 1. Two-variable T test assuming unequal variances yielded *p* < 0.05.

Neither HUSEC 19 nor HUSEC 31 express an O-antigen (Table 1), which leads to their O-rough (OR) phenotype. This phenotype is caused by mutations in genes involved in O-antigen synthesis or assembly, such as *rfb*, *galF*, or *wzy*, reducing virulence and increasing susceptibility to host defenses. Although both HUSEC 19 and HUSEC 31 contain prophages that direct the expression of Stx1, HUSEC 31 produces four-fold more Stx1 than does HUSEC 19. Consistent with previous results [56,63,69], amoeba formed plaques on both of these strains, showing that OR bacteria function as food for *Acanthamoeba*. However, amoebae formed significantly larger plaques (*p* < 0.05) on lawns of HUSEC 19 than they did on HUSEC 31 (Figure 1). This observation shows that HUSEC 31 is more resistant to *A. castellanii* predation than HUSEC 19, suggesting that the elevated level of Stx1 produced by HUSEC 31 plays a significant role in the predation resistance of this strain.

### 3.1. Effect of Stx Type on A. castellanii Predation

**Figure 2:**
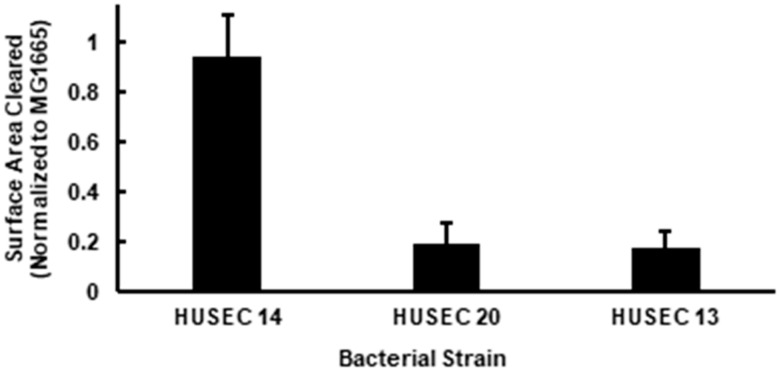
Normalized values for plaque surface area formed by *A. castellanii* on lawns of HUSEC 14, HUSEC 20, and HUSEC 13. All strains express an O26 antigenic serotype and approximately the same induced Stx titer. HUSEC 14 contains phages encoding both Stx1 and Stx2, while HUSEC 20 and HUSEC 13 each express only on toxin type (Stx2 and Stx1, respectively). Two-variable T test assuming unequal variance yielded *p* < 0.05.

HUSEC 14, HUSEC 20 and HUSEC 13 all express the O26 O-antigen (Table 1). We found that amoeba formed plaques on each of these strains, indicating that the O26 serogroup also is recognized and consumed by *Acanthamoeba* (Figure 2). HUSEC 14 and HUSEC 20 express the same total amount of Stx, but HUSEC 14 contains two prophages, one that encodes Stx1 and another that encodes Stx2, whereas HUSEC 20 contains only an Stx2-ecoding prophage (Table 1). We found that amoebae formed much smaller plaques on HUSEC 20 than on HUSEC 14 (Figure 2, *p* < 0.05). This observation suggests that Stx1 and Stx2 may have a differential ability to intoxicate *Acanthamoebae*. Specifically, Stx2 may have stronger anti-predator effects than Stx1.

To test this idea, we compared the size of the amoeba plaques formed on HUSEC 13 and HUSEC 20. HUSEC 13 only bears a prophage that encodes Stx1 and produces only slightly more total Stx than does HUSEC 14 or HUSEC 20. We found that the size of the *Acanthamoeba* plaques formed on HUSEC 13 were significantly smaller that those formed on HUSEC 14 (Figure 2, *p* < 0.05), but similar in size as those formed on HUSEC 20. These findings indicate that Stx1 and Stx2 are both toxic to amoebae (Figure 2). However, a direct comparison of the relative toxicities of Stx1 and Stx2 will require acquisition and comparison of strains that express equal amounts of the two toxin types.

At this point, it is unclear why HUSEC 14 displays only weak resistance to amoebae predation. However, the presence of multiple Stx prophages within a strain have been shown to interfere with each other’s lytic growth [70], suggesting that it is possible that HUSEC 14 may not produce as much Stx1 and/or Stx 2 during *A. castellanii* predation, despite producing similar amounts of total Stx upon exposure to artificial inducing agents (Table 1). Regardless, it is clear that the single Stx-encoding prophages in HUSEC 20 and HUSEC 13 confer a greater survival advantage to these bacterial strains that do the two Stx-encoding prophage found in HUSEC 14.

### 3.2. Effect of O-antigen Type on A. castellanii Predation

**Figure 3:**
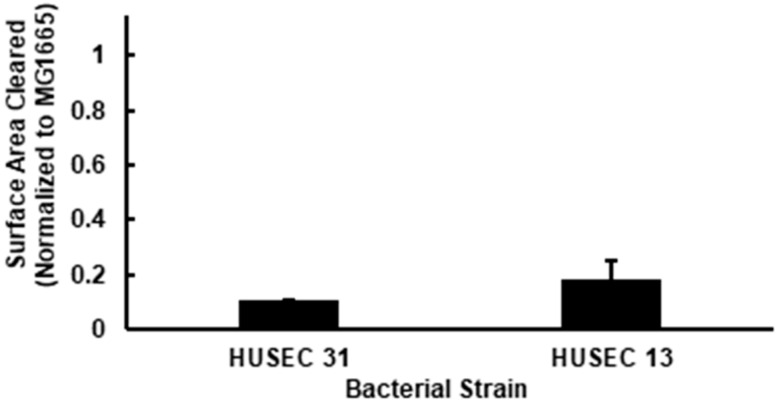
Normalized values for plaque surface area formed by *A. castellanii* on lawns of HUSEC 31 and HUSEC 13. Both strains produce the same amount of Stx1, but HUSEC 31 has an OR serotype, while HUSEC 13 has an O26 serotype. Two-variable T test assuming unequal variance yielded *p* > 0.05.

To help discern the role of O-antigen type in determining STEC predation resistance, we compared the ability of *A. castellanii* to consume HUSEC 31 and HUSEC 13. These strains both bear a Stx1-encoding prophage and produce similar amounts of this toxin. However, HUSEC 31 is an OR strain, whereas HUSEC 13 bears the O26 O-antigen (Table 1). We found no significant difference in the size of the plaques formed on these two strains by *Acanthamoebae* (Figure 3, (*p* ≥ 0.05)). These findings indicate that in a solid phase assay, differences in O-antigens between bacteria that produce the same type and amount of Stx do not play a significant role in mediating anti-predator response to *A. castellanii*. Taken together, our data suggest that the more important factors in this interaction are the Stx type and the amount of Stx produced by the bacteria.

### 3.3. A. castellanii Killing in Liquid Culture

**Figure 4:**
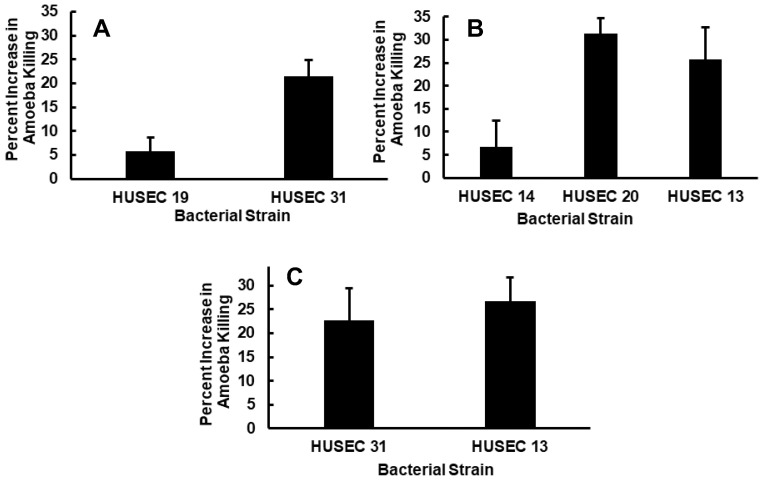
Efficiency of amoebae killing by STEC strains. *Acanthamoebae* were separately co-cultured with each of the STEC strains for 2 h and results are expressed as the percent increase in dead amoeba cells in the presence of STEC bacteria relative to the number of such cells incubated in culture medium alone. (**A**) HUSEC 19 and HUSEC 31 both have the O-rough (OR) serotype, but HUSEC 31 expresses more Stx1. (**B**) HUSEC 14, HUSEC 20 and HUSEC 13 have an O26 serotype and express approximately the same induced Stx titer, but HUSEC 14 contains a phage that encodes for both Stx1 and Stx2, whereas HUSEC 20 and HUSEC 13 contain a single prophage encoding Stx2 or Stx1, respectively. (**C**) HUSEC 31 and HUSEC 13 produce the same amount of Stx1, but have different serotypes (O26 and OR, respectively). Two-variable T test assuming unequal variance yielded *p* > 0.05.

An underlying assumption of the above experiments (Figure 1, Figure 2 and Figure 3) is that the differences in predation resistance result from differences in Stx-mediated amoeba killing, which requires that the amoeba consume the bacteria. However, some additional aspect(s) of the bacteria could prevent or enhance bacterial uptake by amoeba to affect plaque formation by amoebae grown on lawns of STEC bacteria, meaning that differences in plaque size may not reflect actual differences in STEC anti-predator mediated toxicity. To control for this, we directly examined the ability of bacteria to kill *A. castellanii* in axenic liquid culture [63] (Figure 4). In this assay, amoeba viability is directly monitored using Acridine Orange/Propidium Iodide staining.

The results shown in Figure 4A–C mirror those shown in Figure 1, Figure 2 and Figure 3. That is, strains on which amoebae form smaller plaques also kill amoebae at higher efficiencies in liquid culture (i.e., HUSEC 31, HUSEC 20 and HUSEC 13, *p* ≤ 0.05). Further, whereas strains on which amoebae form large plaques (i.e., HUSEC 19 and HUSEC 14) similarly kill amoebae at much lower efficiencies in liquid culture. For example, amoebae form smaller plaques on HUSEC 31 as compared to HUSEC 19 (Figure 1) and HUSEC 31 kills amoebae >3.7-fold more efficiently in liquid culture than does HUSEC 19 (Figure 4). These data suggest that the small plaque sizes observed for HUSEC 31, HUSEC 20 and HUSEC 13 in Figure 1, Figure 2 and Figure 3 are not simply due to the amoeba “ignoring” and not ingesting the bacteria, but instead due to the direct killing of the amoeba by Stx1 and/or Stx2. The results shown in Figure 4 also confirm that Stx type and amount, but not bacterial serogroup, govern the ability of the examined HUSEC strains to resist predation by *Acanthamoebae*. We conclude that STEC resistance to predation is due to Stx-mediated killing of amoebae.

### 3.4. STEC Killing of C. elegans

**Figure 5:**
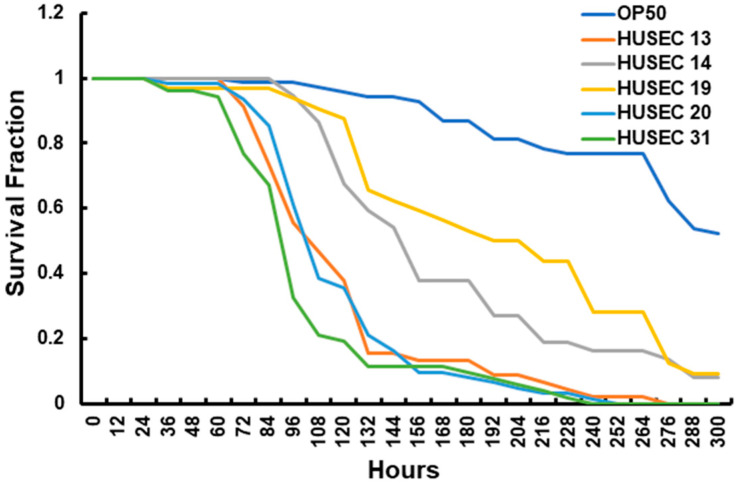
Effects of STEC strain type on the lifespan of *C. elegans*. Wild-type N2 nematodes were placed on plates spread with cultures of the indicated *E. coli* strain. The survival fractions were scored daily. Three independent assays were performed. A representative survival plot is shown.

Having investigated the impact of Stx type/amount and STEC serogroup in governing bacterial resistance to *Acanthamoeba* predation, we assessed the role of these factors on bacterial predation by a complex bacterivore, *C. elegans*, as a model for bacterial defense strategies against multicellular eukaryotic metazoans. *C. elegans* is a free-living nematode that lives in temperate soil environments, feeding almost exclusively on the bacteria that grow in decaying plant matter [71]. For these assays, we examined the same five HUSEC strains (Table 1) on *C. elegans* survival. The *stx*^−^ *E. coli* strain OP50, which is the standard food source used for maintaining *C. elegans* in the laboratory, served as a control for the typical death rate of the worms. Similar to their effects on amoebae (Figure 1, Figure 2, Figure 3 and Figure 4), all of the HUSEC strains significantly decreased *C. elegans* survival when compared to OP50 (Figure 5, Table 2), suggesting that Stx produced by the HUSEC strains kills *C. elegans*.

We found that the restricted mean survival time (RMST) of *C. elegans* feeding on the low Stx1-expressing OR serogroup strain HUSEC 19 was ~225 h, compared to ~198 h for the four-fold-higher Stx1-expressing OR antigen strain HUSEC 31 (Bonferroni *p* value < 2 × 10^−6^). Thus, the higher Stx1 levels led to a ~27 h decrease in RMST, consistent with the effect of these STEC strains on amoeba viability (Figure 4). As described above, the O26 antigen-expressing strains HUSEC 14 and HUSEC 20 produce the same total amount of Stx, but HUSEC 14 contains two prophages, one that encodes Stx1 and another that encodes Stx2, whereas HUSEC 20 only contains an Stx2-encoding prophage. The O26 strain HUSEC 13 bears a prophage that encodes Stx1 but produces more total Stx than does either HUSEC 14 or HUSEC 20. The RMSTs of *C. elegans* grown on HUSEC 20 and HUSEC 13 were essentially identical (~205 h, Bonferroni *p* value = 1.0), whereas the RMST of worms feeding on the O26 strain HUSEC 14 was significantly longer (~221 h, HUSEC 14 vs. HUSEC 20 and HUSEC 14 vs. HUSEC 13 Bonferroni *p* value < 0.02). Thus, the effects of HUSEC 14, HUSEC 20 and HUSEC 13 on *C. elegans* survival mirror their effects on amoeba survival (Figure 2, Figure 3 and Figure 4). While it remains unclear why HUSEC 14 has a smaller effect than does HUSEC 20, and why HUSEC 20 and HUSEC 13 have similar effects against both *A. castellanii* and *C. elegans*, our data suggest that similar mechanisms govern their differential pathogenicity across species (see Section 4). Further, consistent with the similar effects of HUSEC 13 and HUSEC 31 on amoeba survival (Figure 3 and Figure 4), we found no significant difference in the survival curves of *C. elegans* cultured on these strains (Figure 5, Bonferroni *p* value = 1), which differ in O-antigen (O26 versus OR, respectively) but not Stx1 levels (Table 1). This observation further indicates that the absence or presence of the O26 O-antigen does not play a significant role in the pathogenicity of these STEC strains.

## 4. Discussion

### 4.1. Shiga Toxin as a Dual-Use Ecological Weapon

Stx is understood to be a key virulence factor in human pathology, responsible for severe outcomes such as HUS [72]. However, it is unlikely that the Stx-encoding prophage evolved to target human hosts. Instead, our experimental results comparing the survival and killing efficiencies of STEC strains in two distinct eukaryotic predator models—the bacterivorous protist *A. castellanii* and the nematode *C. elegans*—highlight an ecologically relevant role for Stx. Our findings strongly support the hypothesis that Stx initially arose as an anti-predator defense mechanism in the natural environment of bacteria [49,56]. Thus, we propose that Stx production is a “dual-use trait” because it both leads to anti-predator fitness gains (as shown in amoeba and nematodes) and also causes significant disease in the context of accidental hosts like humans [63]. This characterization is also supported by the findings that Stx serves as an anti-predator defense against bacterial predation by *Tetrahymena* [49,73,74].

In the case of unicellular eukaryotic predators such as *A. castellanii* [56,75], the mechanism of intoxication is the established ‘Trojan Horse’ model [56]. In this paradigm, phagocytic predators internalize bacterial cells as a food source, but the stress associated with the phagosomal environment (e.g., oxidative stress) induces the lytic cycle of the Stx-encoding prophage [76]. Phage-mediated lysis of the bacteria leads to the release of high concentrations of Stx [70] directly into the predator’s cytoplasm. This mechanism bypasses the need for the toxin’s canonical cell surface receptor, globotriaosylceramide (Gb3), for entry into cells. But, while the ‘Trojan Horse’ model describes how Stx can enter and kill phagocytes like amoeba, it is not yet clear how Stx intoxicates the non-phagocytic grazer *C. elegans* [51]. Evidence suggests that Stx would be released into the *C. elegans* intestinal lumen following lysis of ingested bacteria [52] and thereafter target specific intestinal cells. However, Gb3 has not been reported in *C. elegans*. Regardless, similar to the case with Gb3-containing mammalian cells undergoing Stx-induced ribotoxic stress response [77,78], exposure of *C. elegans* to STEC O157:H7 activates the conserved p38 MAPK pathway, which is part of the evolutionarily conserved innate immune response in host cells [51]. Irrespective of precise intoxication mechanism, the similarity in killing effectiveness of the STEC strains in these two organisms, using fundamentally different routes of exposure and intoxication, highlights Stx-mediated toxicity as a surprisingly robust, generalist anti-predator defense strategy. This maximization of Stx as an anti-predator molecule may serve to ensure bacterial fitness across diverse ecological niches, including soil, water, and the gastrointestinal tract of ruminant hosts [75].

### 4.2. Titer-Fitness Gradient and the Molecular Determinants of High Titer

The comparative study of isogenic Stx-producing strains demonstrated that anti-predator fitness is not simply a qualitative trait (Stx presence) but is strongly dependent on the quantity of toxin produced. Specifically, the high-titer strain O26 strain (HUSEC 31) consistently exhibited superior killing efficiency in both predator models compared to the lower-titer O26 strain (HUSEC 19) (Figure 1 and Figure 4A). This difference establishes a clear dose-dependent relationship between the amount of Stx released and the resulting predator mortality, suggesting the need for bacteria to develop higher levels of anti-predator fitness and, therefore, efficient phage regulation leading to maximal toxin production.

The variability in Stx titer among STEC strains is rooted in the fine-tuned regulatory dynamics of the integrated lambdoid prophage [79]. High-level production and release of Stx is strictly coupled to prophage induction and later phage-mediated host cell lysis [70]. The efficiency of this lytic switch dictates the final Stx titer. The greater Stx titer of HUSEC 31 strains compared to HUSEC 19 strains is, therefore, likely achieved through genomic variations in the prophage regulatory cascade. The Stx genes are located in the late regulatory region of the prophage, controlled by the late promoter that is regulated by the activity of the Q-antiterminator protein [70]. Multiple phage and host-encoded protein factors impact the frequency and efficiency of prophage induction [80]. The most conserved phage genes that affect induction include the prophage repressor (*cI*) gene [81,82], the Q-protein itself [63], and more recently discovered phage auxiliary genes that increase induction efficiency by affecting host functions [80]. Recent reports also determined that the *exo-xis* region of the prophage genome plays a role in the regulating lysogenization and prophage induction [83,84,85]. Metabolic status also affects prophage induction frequency [86].

Analysis of the genomic sequences of the prophages in HUSEC 19 and HUSEC 31 indicates that the sequences of the putative *cl* genes in both prophages are highly similar, but the sequences of the putative Q-proteins are more divergent. We also note that the sequence of the putative Q-protein of HUSEC 13, which produces the same amount of Stx as does HUSEC 31, is identical to that of HUSEC 19. Thus, it is as yet unclear what factors control the differential production of Stx in the HUSEC strains we examined. However, it is well known that the induction of a given prophage can be modulated by interactions with other resident prophages [87]. Recent evidence shows that phages employ cross-regulatory strategies, where the repressor of the phage can bind to and inhibit the lytic genes of co-hosted prophages [88]. This interference conserves host cell resources for the optimal replication of the phage, preventing the production of competing virions and thereby maximizing yield and later killing power [88].

The regulatory efficiency that leads to high toxin titer is also intimately linked to the host’s metabolic state. Carriage of the phage profoundly affects *E. coli* carbon metabolism, leading to a reprogramming that favors mixed acid fermentation over aerobic respiration [86]. This metabolic shift is crucial for fitness in the anaerobic, nutrient-limited environment of the ruminant gut or within a predator’s phagosome. High-titer strains may benefit more from enhanced respiration with sugar components of intestinal mucus, such as arabinose, fucose, and N-acetyl-glucosamine. This metabolic pre-adaptation enhances the persistence and survival of the bacterial population within the reservoir of the predator itself, increasing the total period available for localized phage induction and sufficient accumulation. If correct, this tightly integrated link between regulatory efficiency, metabolic fitness, and ecological potency would constitute a strong evolutionary selection target for toxin production.

### 4.3. Impact of Toxin Type on Predation Resistance

Stx1 and Stx2 share structural and enzymatic characteristics. However, they differ in sequence, biological activity and serological reactivity [3]. The two Stx subtypes are epidemiologically associated with different clinical outcomes after STEC infection, with Stx2 subtypes more frequently associated with a higher risk of developing HUS. The robust toxicity of the HUSEC strains to *A. castellanii* and *C. elegans* aligns with findings that STEC O157:H7 also successfully kills the nematode host [51]. However, these investigators report that in the context of O157:H7, only Stx1-expressing strains appeared to significantly affect the survival of *C. elegans*. Strains expressing only Stx2 had almost no effect on *C. elegans* lifespan. In contrast, our results indicate that both Stx1 and Stx2 anti-predator activities are similar irrespective of the predator model (Figure 4 and Figure 5). To help resolve the discrepancy between the two studies, we note that Chou et al. [51] found that beyond the toxin itself, the ability of STEC O157:H7 to colonize the nematode intestine, induce Attaching and Effacing (A/E) lesion, and cause microvillar damage all contributed to overall nematode virulence. Furthermore, Youn et al. [52] showed that the large virulence plasmid pO157 is also required for effective killing of *C. elegans* by STEC O157:H7. Thus, it seems that O157:H7 may represent a specialized case wherein the evolution of this strain to more effectively kill humans limits the ability of Stx2 to function as an anti-predator defense molecule. This suggestion is consistent with our findings showing that more recently evolved STEC O157:H7 bacteria kill amoebae less efficiently than progenitor clades [63]. In aggregate, these collective findings confirm that the efficiency of bacterial killing of predators is a multi-factorial trait involving Stx amount, type and other host factors.

### 4.4. Role of the Bacterial Cell Surface in Predation Resistance

Successful Stx deployment is preceded by the requirement for the bacterium to be internalized and survive within the predator for a sufficient period to induce the phage and produce Stx. Bacterial recognition and uptake efficiency are dictated by the composition and structure of the bacterial cell surface, particularly the lipopolysaccharide (LPS). For example, we found that the LPS oligosaccharide (OS) region modulates bacterial recognition by *A. castellanii* [69]. Despite the importance of cell surface interactions in microbial predator/prey interactions, we found that strains producing identical amounts of Stx1, but which bear different O-antigens, display similar killing efficiencies in the context of both *A. castellanii* and *C. elegans* (Figure 3, Figure 4A and Figure 5). This result indicates that O-antigen, at least in the context of O26:H11 (HUSEC 13) versus O-rough (HUSEC 31), does not play a significant role in mediating the anti-predator response to either *A. castellanii* or *C. elegans*. This is an important distinction from studies on other virulence factors. For instance, in *E. coli* O157:H7, pO157 and LPS O-side chains are required for killing *C. elegans* [52], demonstrating that for some, but not all STEC strains, non-toxin factors are crucial for successful host colonization and pathogenicity. Although our investigations probed a limited set of Stx-encoding strains, our findings strongly support the conclusion that the Stx type and amount are the more important factors in these specific predator–prey interactions.

### 4.5. STEC Fitness and Virulence

The convergence of findings from ecological predation models and epidemiological data represents the most significant conclusion of this research: ecological selective pressure imposed by protists likely acts as a strong evolutionary filter, selecting for Stx-producing strains optimized for defense, that are inadvertently virulent in the human host [63,75]. Evolutionary models substantiate this phenomenon, predicting that intense predation pressure can shift the evolutionarily stable virulence towards more severe strains compared to predator-free systems [89]. This study successfully supports and refines the ecological model of STEC virulence evolution. That is, high anti-predator fitness enables the persistence of the STEC clone in the natural environment. Significantly, we note that the trait selected for maximal fitness against environmental predators (high titer) is precisely that which corresponds to the highest risk factor for severe human disease, demonstrating a powerful evolutionary link.

## 5. Conclusions

This study demonstrates that variation in Shiga toxin (Stx) type and production level are key determinants of the ability of Shiga toxin-producing *Escherichia coli* (STEC) to resist predation by eukaryotic bacterivores. Importantly, the patterns of predator killing observed across the STEC strains parallel epidemiological associations between toxin production and disease severity in humans. Strains producing higher toxin titers or harboring particular toxin types displayed enhanced toxicity toward both model predators, supporting the concept that Stx functions as a “dual-use” ecological weapon. Traits that increase bacterial fitness in environmental predator–prey interactions may therefore inadvertently generate strains with elevated virulence potential in accidental hosts such as humans.

These findings strengthen the ecological model for the evolution of STEC virulence and highlight bacteriophage-regulated toxin production as a critical determinant of cross-species pathogenicity. Future work should focus on identifying the molecular mechanisms that control differences in Stx expression among prophages and determining how additional bacterial or phage-encoded factors influence toxin deployment in natural environments. Understanding these regulatory pathways may ultimately provide new strategies for predicting, monitoring, or mitigating the emergence of highly virulent STEC strains.

## Figures and Tables

**Table 1:** Five HUSEC (hemolytic uremic syndrome)-associated *E. coli* ([57]) strains selected for these experiments based on Stx type, Stx titer, and serotype.

Strain	Serotype	Stx Type	Stx Titer *
HUSEC 13	O26:H11	Stx1	724
HUSEC 14	O26:H-	Stx1 and 2	512
HUSEC 19	OR:H11	Stx1	181
HUSEC 20	O26:H11	Stx2	512
HUSEC 31	OR:H-	Stx1	724

* The Stx titer was determined using the Vero cell assay and serial 1:2 dilutions of the bacterial supernatant needed to kill the mammalian cells as described in [58,59].

**Table 2:** The effects of the five HUSEC (hemolytic uremic syndrome)-associated *E. coli* ([57]) strains on *C. elegans* lifespan are indicated as restricted mean survival time (RMST), given as hours.

Bacterial Strain	Restricted Mean Survival Time Hours (± Std Error)
OP50	239.4 (3.4)
H13	204.9 (2.3)
H14	221.2 (2.6)
H19	225.3 (3.3)
H20	205.6 (1.9)
H31	198.2 (2.2)

## Data Availability

The original contributions presented in this study are included in the article. Further inquiries can be directed to the corresponding author.

## Abbreviations

The following abbreviations are used in this manuscript:

HUS	Hemolytic uremic syndrome
Stx	Shiga toxin
STEC	Shiga toxin-producing *E. coli*
